# All-Optical Tuning Based on Magnetic Fluid-Filled Microcapillary Resonators Inserted with Half-Cone Fiber

**DOI:** 10.3390/s25061784

**Published:** 2025-03-13

**Authors:** Minggang Chai, Caijun Xue, Mengyu Wang, Yanjun Fu, Chengfeng Xie

**Affiliations:** 1 College of Aerospace Engineering, Nanjing University of Aeronautics and Astronautics, Nanjing 210016, China; mgchai@nchu.edu.cn; 2 Key Laboratory of Nondestructive Test (Ministry of Education), Nanchang Hangkong University, Nanchang 330063, China; mengyu@nchu.edu.cn (M.W.); 23021@nchu.edu.cn (Y.F.); 3 Key Laboratory for Optoelectronic Information Perception and Instrumentation of Jiangxi Province, Nanchang Hangkong University, Nanchang 330063, China

**Keywords:** microresonator, tuning, whispering-gallery modes, microcapillary, power sensor

## Abstract

In this study, we designed and experimentally demonstrated an all–optical tuning system based on the absorption effect of magnetic nanoparticles on a pump light. The all-optical tuning process induces a temperature change in the microcavity–taper coupling system, resulting in a shift in the WGM resonance spectrum. The core of the sensor involved in this study is a microcapillary resonator with a microfluidic channel, in which a magnetic fluid is filled within the channel of the microcapillary resonator. We tested the sensing sensitivity of microcapillary resonators with two sizes. The experimental results indicate that for the larger microcapillary resonator, the sensitivity is 0.0347 nm/mW when the pump light power increases, and 0.0331 nm/mW when the pump light power decreases. For the smaller microcapillary resonator, the sensitivity significantly increases, with 0.1018 nm/mW and 0.1029 nm/mW as the power increases and decreases, respectively. The demonstrated optofluidic device has the advantages of small size, good repeatability, high sensitivity, and low price, and thus shows great potential for sensing applications.

## 1. Introduction

Optical microresonators supporting whispering-gallery modes (WGMs) are promising for high–sensitivity sensing applications [1]. This is because they can confine light to a small space, which greatly enhances the interactions between light and matter. Compared with conventional optical resonators, WGM microresonators have a very high quality (*Q*) factor and a very small mode volume [2]. WGM optical microresonators can also take various shapes, such as spherical, bottle, and disk shapes [3,4]. Microcapillary resonators, in particular, with their natural fluid channels, confine WGMs in the walls of the tubes through continuous total internal reflection on their surfaces. The symmetric structure of these resonators can excite rich mode spectra, enhancing the binding effect of light, as well as the interaction between the abruptly passing light field and the substance being measured [5]. As a result, microcapillary resonators have potential applications in biochemical analysis and detection, as high-sensitivity sensors, and in nonlinear optics [6,7,8].

A microcapillary resonator is one kind of optofluidic ring resonator [9,10]. Microcapillary resonators combined with microfluidic techniques have a highly sensitive optical part and exploit the unique properties of liquid phase materials, such as their active (providing gain) and absorbing properties. Moreover, because liquid phase materials are fluidic and displaceable, they provide many unique properties that enhance sensing performance and simplify microsystem design. As a result, optofluidic microcapillary resonators have received considerable attention from researchers due to their wide potential applications.

Research has shown that tunable optical flow microcapillary resonators can be achieved by displacing the liquid phase material in the system or by changing the concentration of the liquid phase material. In 2006, White IM et al. demonstrated an all–optical tunable optical flow microcapillary resonator based on micro–nano fiber cone-coupled microcapillary resonators, achieving 2.6 nm/RIU and 10 nm/RIU (Refractive Index Units) detection sensitivity [11]. In 2009, Sun Y et al. demonstrated fast detection of dinitrotoluene vapor at room temperature using an optical flow–controlled microcapillary resonator sensor, which is particularly useful for detecting explosives in real, complex vapor samples [12]. In 2013, Kim KH et al. performed the first optical–mechanical fluid interaction study, opening up new research directions for using cavity optomechanics to study non-solid-phase materials [13]. In 2019, David C et al. reported on the specific detection of cadmium ions in water using an optofluidic sensor based on a polymeric microcapillary resonator functionalized with a specific ligand for cadmium ions. This chemical sensor can be used to build portable field analytical instruments [14]. In 2022, Niu P et al. proposed a disposable optofluidic microcapillary immunosensor for label–free cardiac troponin complex detection, which could serve as an innovative detection method for early diagnosis of myocardial infarction [15].

In this paper, we demonstrate all–optical tuning of magnetofluid–filled microcapillary resonators. We study the parameters of two sizes of microcapillary resonators and compare their characteristics in optical flow–tuning sensing. Firstly, we prepare high–*Q* microcapillary resonators using the arc discharge method and test the resonant characteristics of the two sizes using a broadband light source as the excitation source. Secondly, we use the input end of the half–cone fiber to connect to the pump light source, and use the fiber tip as a point heat source. The temperature increase generated by the strong photothermal effect of the magnetic fluid causes WGM spectral drift, leading to a change in the effective refractive index of the coupling system. Finally, we measure the resonant wavelength characteristics of the two sizes of microcapillary resonators with power and investigate the relationship between the resonant wavelength shift and absorbed power of the two sizes. This work opens up a new way for the application of all–optical devices.

## 2. Theoretical Basis

**Figure 1:**
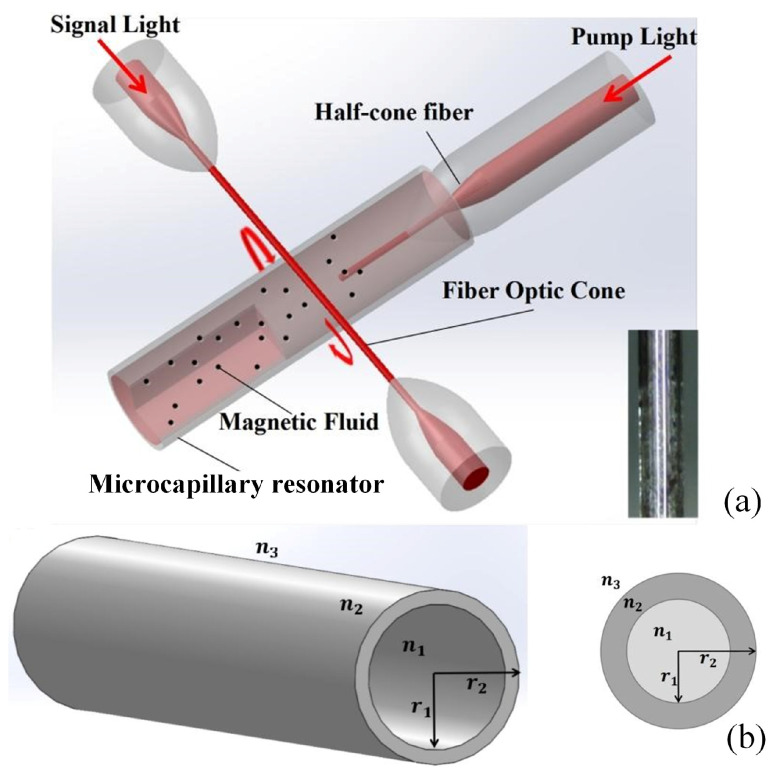
(**a**) Schematic diagram of an all-optical tunable microcapillary resonator with black dotted particles dispersed in a brick-red carrier fluid, representing magnetic nanoparticle particles in a magnetic fluid. The inset shows a microcapillary resonator filled with magnetic fluid. (**b**) Schematic diagram of the structure of the microcapillary resonator. Section of the microcapillary resonator. $n_{1},n_{2}{,n}_{3}$ denote the refractive index of the medium inside the tube, the tube wall, and the material of the ambient medium, and $r_{1}$ and $r_{2}$ are the inner and outer radii of the microcapillary resonator.

The object of this study is the microcapillary resonator, which is a hollow tube structure made of silica fibers. It can also be referred to as a hollow fiber or a liquid–core fiber. Unlike conventional optical fibers, the microcapillary resonator contains air or liquid instead of having a core [11,16]. The structure can be seen as a double-layer radial structure, as shown in Figure 1. In a microcapillary resonator, light is coupled into the wall and circulated along the circumference; when the incident light interferes with the propagating light in the microcapillary resonator at phase length, the resonant wavelength can be expressed by the following equation [17]:

(1) $$2\pi rn_{eff}=m\lambda$$

where λ is the resonant wavelength, m is the angular mode number, r is the outer radius of the resonant cavity of the microcapillary, and $n_{eff}$ is the effective refractive index (RI) experienced by the circulating light. Based on the double-layer structural model of the microcapillary resonator, the resonant wavelength is jointly determined by the refractive indices of the core (filling material), the tube wall, and the ambient medium (e.g., air). Under the thermo–optical and thermal expansion effects [18], the temperature affects the resonance characteristics of the microcapillary resonator, causing the WGM resonant wavelength to drift, which can be expressed by the following equation [19]:

(2) $$\frac{\Delta \lambda }{\lambda }=\alpha \Delta Τ+\frac{\partial n_{eff}}{\partial n_{2}}\frac{\kappa_{wall}}{n_{eff}}\Delta T+\frac{\partial n_{eff}}{\partial n_{1}}\frac{\kappa_{core}}{n_{eff}}\Delta T$$

where $\alpha =\left(\partial r/\partial T\right)\cdot \left(1/r\right)$ is the thermal expansion coefficient of the microcapillary resonator and $\kappa_{wall\left(core\right)}={\partial n}_{wall\left(core\right)}/\partial T$ represents the thermo-optical coefficient of the tube wall and core, respectively. The geometric parameters of the microcapillary resonator are fixed. The thermal expansion of r caused by the temperature T is negligible due to its relatively thick wall, so the temperature T is considered the main factor affecting the effective refractive index $n_{eff}$. Then, the relationship between temperature T, $n_{eff}$, and resonance shift ∆λ can be expressed as [20]

(3) $$\Delta \lambda =\frac{\lambda }{n_{eff}}\cdot \frac{\partial n_{eff}}{\partial T}\cdot ∆T$$

where $∆T=Μ\cdot P_{pump}$ is the temperature variation, $P_{pump}$ is the pump optical power injected directly into the magnetic fluid inside the microcapillary resonator, Μ is the correlation coefficient, which is related to the absorption coefficient of the magnetic fluid (${Fe}_{4}O_{3}$) and the thermal conductivity and geometric parameters of the microcapillary resonator [21], and $\partial n_{eff}∕\partial Τ$ is positively correlated with the thermo–optical coefficient of the material silica. The equation reflects that the pump optical power can effectively control the resonance displacement, while the structural parameters and properties have been determined when preparing the device and selecting the material, so the resonance shift ∆λ depends only on the pump power $P_{pump}$. It can be deduced that ∆λ increases approximately linearly with the increase of $P_{pump}$ in a certain range.

## 3. Materials and Devices

The conventional method for preparing microcapillary resonators involves heating and melting commercial silica capillaries [22], followed by motor drawing to make them thinner. The microresonators are then etched using hydrofluoric acid to produce microcapillary resonators with different wall thicknesses, which can be controlled by adjusting the etching time. However, in the current study, it was not necessary to further reduce the wall thickness, since a sufficiently large thickness is required to confine the majority of light within the wall and prevent significant absorption of signal light at resonant wavelengths by the magnetic fluid.

**Figure 2:**
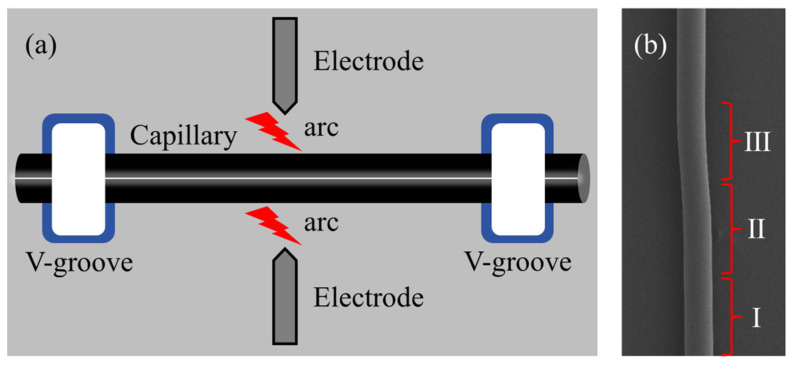
(**a**) Schematic diagram of high-*Q* microcapillary resonator processing. (**b**) Microscopic image of microcapillary resonators; regions I, II, and III represent normal smooth region, super smooth region, and depressed region, respectively; arrows point to the coupling position between fiber cone and microcapillary resonators.

To begin, a section of capillary tube is taken (outer radius: 161 μm/80 μm, inner radius: 128 μm/58 μm, wall thickness: 33 μm/22 μm, Ronglipu Scientific Instrument Co. Ltd., Jiangsu, China) and the coating layer on the outside of the tube is burned off with a hydrogen–oxygen flame from the fusion cone drawing machine. The tube is then wiped clean with alcohol. The processed capillary tube is fixed in two V–shaped grooves of the fiber optic fusion splicer (Fujikura FSM–40S) between the middle two electrodes, as shown in Figure 2a. By using the arc discharge and traction force of the fiber optic fusion splicer, the capillary tube structure is made concave. The degree of concavity is adjusted by regulating the discharge current and the number of discharges. The microcapillary resonator is divided into three regions (I, II, III), representing the normal smooth region, the super smooth region, and the depressed region, as shown in Figure 2b. Due to the melting and settling of the fiber material caused by the discharge operation, region II has an ultra-smooth surface. Only the ultra–smooth region (II) can support ultra–high *Q* WGMs among these three regions [23].

Lastly, the processed microcapillary resonator is filled with magnetic fluid (Particle radius: 10 nm–30 nm, Macklin Biologics Co. Ltd., Shanghai, China) and sealed at the bottom. A prefabricated fiber with no coating is then stretched into a micro–nano fiber with a diameter of approximately 2 μm [24]. This fiber is cut into two halves, and one section of the half–cone fiber is selected. It is inserted into the fluid channel of the microcapillary resonator to serve as a transmission mediator for pumping optical input, as shown in Figure 1.

## 4. Experimental Results and Discussion

**Figure 3:**
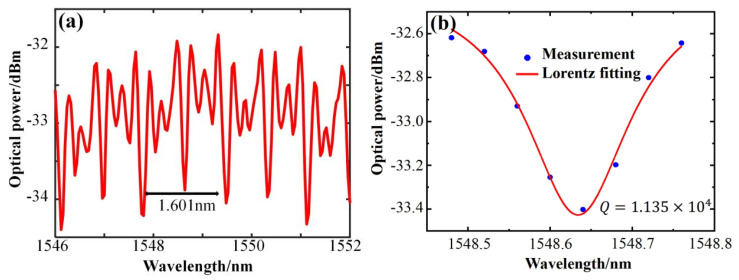

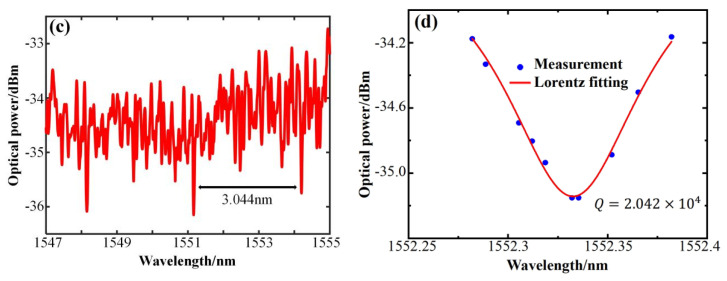
Transmission spectra and resonance peak fitting of microcapillary resonators of two sizes. (**a**,**b**) Transmission spectra and Lorentz fitting of the microcapillary resonators with $r_{1}$ = 128 μm, $r_{2}$ = 161 μm, and wall thickness of 33 μm. (**c**,**d**) Transmission spectra and Lorentz fitting of the microcapillary resonators with $r_{1}$ = 58 μm, $r_{2}$ = 80 μm, and wall thickness of 22 μm.

The quality factor *Q* value is a fundamental characteristic parameter of an optical microcavity. In this study, the resonant spectral characteristics of a large–sized microcapillary resonator with an inner radius of 128 μm, an outer radius of 161 μm, and a wall thickness of 33 μm were tested for the first time. A tapered fiber, with a tapered waist diameter of about 2 μm, was used to excite the resonant spectrum of the microcapillary resonator. Figure 3a displays the spectra obtained by coupling the tapered fiber with the microcapillary resonator. The figure indicates that the resonant mode of the microcapillary resonator can be clearly excited, and the spectral lines are neat and regular. The theoretical value of the free spectra range (FSR) of the microcapillary resonator can be calculated by the formula $∆\lambda_{FSR}=\lambda^{2}/\pi n_{g}r_{2}$, where λ is the resonant wavelength and $n_{g}$ is the group velocity refractive index. To simplify the calculation, the refractive index value of the microcapillary material is used instead. The FSR of the microcapillary resonator of this size is 1.601 nm, and the FSR calculated by the theoretical formula is 1.645 nm, and these are very close to each other. In the analysis of the microcapillary resonator, the *Q* value can be estimated using the equation Q=λ/δλ [25], where λ is the central resonant wavelength and δλ is the half–height full width of the resonant spectrum. Figure 3b is the result of the Lorentz fitting taken from the resonance spectrum of Figure 3a at the central wavelength of the resonant peak near 1548.634 nm. The half–height full width of the resonance spectrum is 0.1365 nm, and the *Q* value of the resonance mode is $1.135\times 10^{4}$ by the above *Q* value calculation formula.

Figure 3c displays the resonance spectra excited by a small–sized microcapillary resonator, with an inner radius of 58 μm, an outer radius of 80 μm, and a wall thickness of 22 μm. Observation of the resonant spectrum obtained by coupling the microcapillary resonator in the ultra-smooth region reveals that more resonant peaks are efficiently excited within one FSR than another size microcapillary resonator, resulting in a dense resonant spectrum. On the one hand, small-sized microcapillaries support a wider range of free spectra, allowing multiple higher–order modes to exist within an FSR interval. In addition, the good phase matching relationship between the tapered fiber and the microcapillary can also excite dense spectral peaks. The theoretical equation predicts an FSR of 3.329 nm, and the experimentally measured FSR is 3.044 nm, which is very close to the theoretical value. Figure 3d shows the results of Lorentz fitting to the WGM resonance spectrum at the center wavelength of the resonance peak of 1552.332 nm. The half–height full width of this mode is 0.076 nm, which leads to a calculated *Q* value of $2.042\times 10^{4}$.

**Figure 4:**
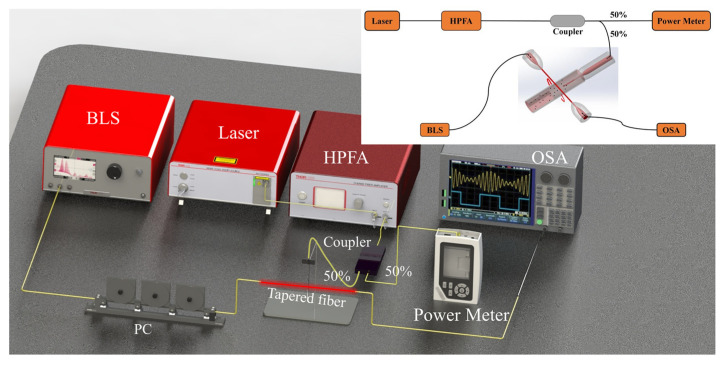
Experimental setup of an all-optical tuned microcapillary resonator. Laser: single frequency fiber laser; HPFA: high–power fiber amplifier; BLS: broadband light source; OSA: optical pulsed analyzer; PC: polarization controller.

Figure 4 shows the experimental setup for the all-optical tuning of the microcapillary resonator. The setup involves placing the micro–nano fiber cone in vertical contact with the ultra-smooth surface region of the microcapillary resonator and using the strong swept field of the micro–nano fiber to couple the light inside the fiber into the microcapillary resonator, thereby exciting the WGMs present within. The output of the micro–nano fiber used for coupling can be measured by a spectrometer that allows for optical transmission with depressions at a specific wavelength spectrum. The internal channel of the microcapillary resonator is designed to hold magnetic fluid. After tunable light injection, the photothermal effects generated by the magnetic particles upon absorbing light energy induce a shift in the resonant frequency of WGMs. For the liquid injection operation, one stem of the microcapillary is connected to a syringe via a rubber hose, and the magnetic fluid prepared in advance is injected into the hose through the syringe, allowing it to flow along the hose and eventually fill the entire space of the microcapillary. The other stem of the microcapillary is also connected to a waste collection pool with a rubber hose to facilitate the fluid recovery after the experiment. In the all–optical tuning experiment, one stem of the microcapillary injected with magnetic fluid is sealed with UV glue, and the other stem is inserted into the prepared half-cone fiber, which acts as a pump light injection tool to control the power of the pump light injected into the magnetic fluid. Due to the strong photothermal effect of the magnetic fluid, the absorption of a small amount of light in a very small range generates a high temperature, causing resonance drift in the resonance spectrum.

**Figure 5:**
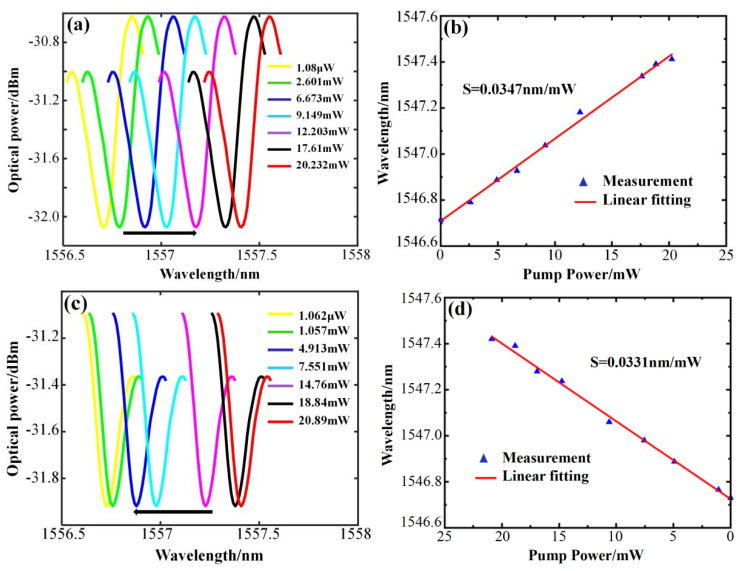
Sensing characteristics and linear fitting of the microcapillary resonators with $r_{1}$ = 128 μm, $r_{2}$ = 161 μm, and a wall thickness of 33 μm. (**a**) Resonance spectrum at increasing power. (**b**) Linear fitting at increasing power. (**c**) Resonance spectrum at decreasing power. (**d**) Linear fitting at decreasing power.

For the experiment, a single–frequency fiber laser serves as the pump light source. The laser generates a continuous beam as the pump light signal, which is amplified by a high-power fiber amplifier and injected into the microcapillary channel through a 1:1 beam splitter via a half–cone fiber. The pump light power is monitored in real time using an optical power meter to control the pump light power. The resonator size is $r_{1}$ = 128 μm, $r_{2}$ = 161 μm, and has a wall thickness of 33 μm. Figure 5 displays the variation in the WGM resonance spectrum of the microcapillary resonator with power, collected as the power increases and decreases, respectively. Figure 5a shows the WGM resonant wavelength shifted toward the long-wave direction when the pump light power rises from 1.08 μW to 33.232 mW, with a red shift of 0.702 nm, and the power sensitivity is obtained as 0.0347 nm/mW by linear fitting as in Figure 5b. Figure 5c shows the WGM resonant wavelength shifts in the short-wave direction as the pump light input power decreases from 33.89 mW to 1.062 μW, with a blue shift of 0.691 nm, and the power sensitivity is 0.0331 nm/mW, obtained by linear fitting as in Figure 5d. The power sensitivities obtained during the power increase and power decrease are very close to each other, with an average sensitivity of 0.0339 nm/mW. The results show good consistency and feasibility.

**Figure 6:**
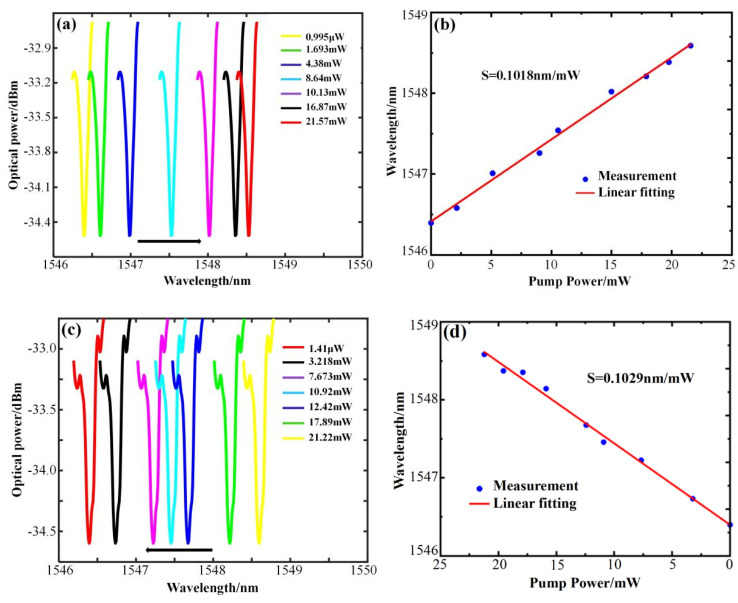
Sensing characteristics and linear fitting of microcapillary resonators with $r_{1}$ = 58 μm, $r_{2}$ = 80 μm, and 22 μm wall thickness. (**a**) Resonant spectra at increasing power. (**b**) Linear fitting at increasing power. (**c**) Resonant spectra at decreasing power. (**d**) Linear fitting at decreasing power.

The sensing characteristics of the microcapillary resonator with $r_{1}$ = 58 μm, $r_{2}$ = 80 μm, and a wall thickness of 22 μm were tested using the same method. The WGM resonance spectra were collected when the power was rising and falling, respectively. Figure 6a shows that when the pump light power rises from 0.995 μW to 21.57 mW, the WGM resonant wavelength shifts toward the long–wave direction by 2.195 nm, and the power sensitivity of 0.1018 nm/mW can be obtained as in Figure 6b. As shown in Figure 6c, when the power decreases from 21.22 mW to 1.41 μW, the WGM resonant wavelength shifts toward the short–wave direction by 2.184 nm, and a power sensitivity of 0.1029 nm/mW can be obtained from Figure 6d. The power sensitivities obtained in the process of power increase and decrease are very close, with an average sensitivity of 0.10235 nm/mW.

Table 1 shows the sensitivity comparison of two sizes of optical microcapillary resonators. In the hollow (air) case, the resonant wavelength shift induced by all–optical tuning is very small and almost negligible, while in the case of filling with magnetic fluid, the sensitivity is two orders higher than that in the hollow case because of the strong absorption effect and photothermal effect of the magnetic fluid, verifying the effect of the magnetic fluid on the spectral performance of the device. Compared to the large–sized microcapillary ($r_{1}$ = 128 μm, $r_{2}$ = 161 μm, and wall thickness of 33 μm), the average sensitivity of all–optical tuning in the small–sized microcapillary ($r_{1}$ = 58 μm, $r_{2}$ = 80 μm, and wall thickness of 22 μm) is increased by three times. This is mainly due to the smaller mode volume and higher mode field energy density, which make the interaction between light field and analytes stronger [26].

**Figure 7:**
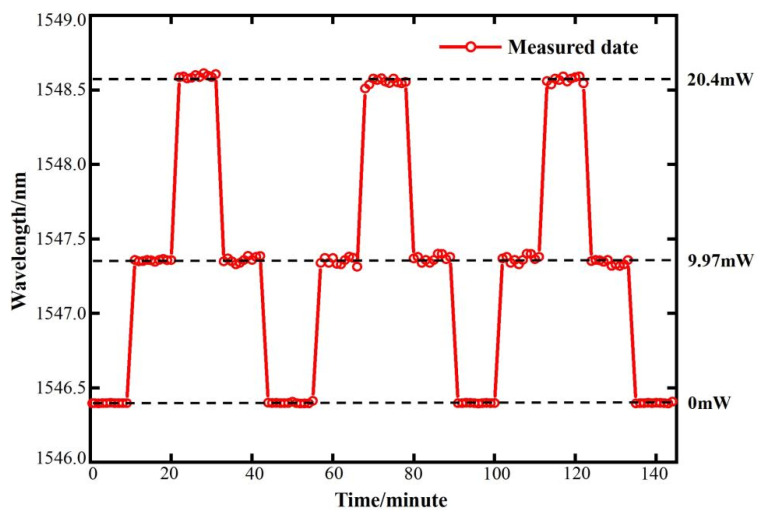
Resonant wavelength variation with time for the sensing system under 0 mW, 9.97 mW, and 33.4 mW pump optical power cycles.

To analyze the spectrum evolution with pump light power over time, resonant modes excited in the small–sized microcapillary resonator were measured. The pump power was set to cycle between 0 mW, 9.97 mW, and 33.40 mW at 10 min intervals. During the experiment, the WGM spectra of the sensor were sampled at fixed time intervals of 60 s, while the power value was monitored by a power meter. Figure 7 displays the variation in the resonant wavelength with time when the power is cycled. The applied pump power is repeated for 145 min under three power cycles, and the WGM spectral displacement in response to power change remains consistent with time, indicating that the sensor is not affected by the repeated power cycles. Small fluctuations observed in the WGM spectral position when the power is elevated may be due to the instability of the high–power fiber amplifier.

**Figure 8:**
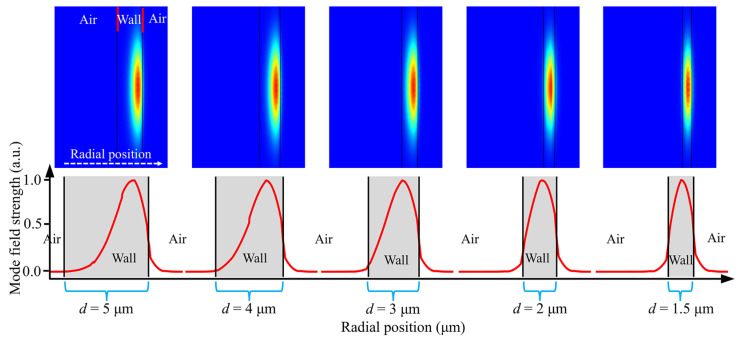
Mode field distributions of the WGMs for various wall thicknesses of microcapillary resonators.

To elucidate the interaction between the WGMs of microcapillary resonators and the material confined within the tube, we conducted further numerical simulations and analysis of the devices. As illustrated in Figure 8, we simulated the electromagnetic field distributions of the WGMs for various wall thicknesses (*d*). The horizontal axis represents the radial position of the microcapillary resonator, and the vertical axis represents the normalized mode field strength. “Wall” represents the wall thickness of the microcapillary, and “Air” represents the air environment. The red curves arranged in sequence depict the WGM field distributions of microcapillaries with different wall thicknesses along the radial direction. Observations reveal that for wall thicknesses exceeding 3 μm (*d* = 4 μm, *d* = 5 μm), the WGMs predominantly localize on the outer side of the tube wall. At a wall thickness of 3 μm, radial WGMs begin to exhibit partial distribution on the inner side of the tube wall, manifesting as evanescent fields within the hollow layer of the tube wall. Conversely, at a wall thickness of 2 μm, the evanescent field within the hollow layer intensifies, resulting in the concentration of WGMs towards the inner side of the tube wall. When the wall thickness *d* = 1.5 μm, the WGMs inside the hollow layer of the tube wall continue to enhance, and the microcapillary resonator modes are concentrated in the direction of the inside of the tube wall. As a result, a stabilized WGM pattern is formed in the axial direction by the formation of a raised structure on the outer side of the tube wall under the action of the arc discharge. Reducing the wall thickness of the microcapillary resonators to below 3 μm leads to a further concentration of WGMs towards the inner side of the tube wall, augmenting the interaction between light and matter and thus enhancing the tuning performance of the microcapillary resonators. Microcapillary resonators prepared conventionally using hydrofluoric acid etching can reduce the wall thickness of the tube. However, it concurrently diminishes the *Q* factor, thereby attenuating the interaction between WGMs and substances within the tube. Our method via arc discharge exhibits highly regular resonance mode spectra and precise alignment with a high*Q*–factor characteristic. We demonstrated the advantages of our proposed technology by comparing some all–optical tuning works, see Table 2. 

The comparison content is presented using a table. For example, reference [28] has a similar tuning sensitivity to our work, but a larger tuning range. However, its sensor core uses a six–hole structure, which increases the interaction area between the Fe_3_O_4_ particles and the WGM field. Additionally, the remaining Fe_3_O_4_ particles after evaporation possess a stronger light–thermal conversion capability. In contrast, our work employs only a single–channel microcapillary and uses a magnetic fluid solution as the all–optical tuning medium, making our device more cost–effective and simplifying the all–optical tuning operation (see Table 2).

## 5. Conclusions

In summary, we have studied the preparation of microcapillary resonators and the sensing characteristics of microcapillary resonators of different sizes in theory and through experiments. We proposed a novel and flexible all–optical tunable optofluidic microcapillary resonator by using the strong absorption effect of magneto–fluid on light and a strong photothermal effect. The sensitivity of the two sizes of microcapillary resonators at power increase and power decrease are characterized, respectively, and the results show that the small-size microcapillary resonator is about three times more sensitive than the large-size microcapillary resonator. The result indicates that the small-size microcapillary resonator’s performance is more advantageous. The tuning sensitivity and tuning range of the sensor were tested at low threshold pumping optical powers, and the highest reached 0.1029 nm/mW and 2.195 nm, respectively, which has obvious advantages compared with other tuning schemes. Meanwhile, the device was proved to have good stability and repeatability through several tests on dynamic performance. Due to its excellent performance, the optofluidic microcapillary resonator has great potential in sensing technology, precision measurement, and bio–detection.

## Figures and Tables

**Table 1:** Comparison of resonant cavity parameters and sensing characteristics of two sizes of microcapillary resonators.

Filling Material	$r_{1}$ (μm)	$r_{2}$	Wall Thickness (μm)	Rise Sensitivity (nm/mW)	Decrease Sensitivity (nm/mW)
Magnetic fluid	128	161	33	0.0347	0.0331
	58	80	22	0.1018	0.1029
Air	128	161	33	0	0
	58	80	22	0.0023	0.0024

**Table 2:** Comparison of different all-optical tuning schemes for WGM resonators.

Sensor	Heating Materials	Sensitivity	Tuning Range
Photonic crystal fiber resonator [27]	Fe_3_O_4_ NPs	0.034 nm/mW	3.6 nm
Grapefruit microstructured fiber resonator [28]	Fe_3_O_4_ NPs	0.106 nm/mW	5.32 nm
Silica microbottle resonator [29]	Fe_3_O_4_ NPs	0.022 nm/mW	0.68 nm
Silica capillary resonator [30]	Fe_3_O_4_ NPs	0.0382 nm/mW	0.15 nm
Our work	Fe_3_O_4_ NPs	0.1029 nm/mW	2.184 nm

## Data Availability

Data are contained within the article.
